# Brain structure and function differences across varying levels of endurance training: a cross-sectional study

**DOI:** 10.3389/fnhum.2024.1503094

**Published:** 2024-11-29

**Authors:** Keying Zhang, Chunmei Cao, Yaxue Wang, Dong Zhang

**Affiliations:** ^1^Department of Physical Education, Southeast University, Nanjing, China; ^2^Division of Sports Science and Physical Education, Tsinghua University, Beijing, China; ^3^Department of Physical Education, Hebei Normal University, Shijiazhuang, China; ^4^Institute of Artificial Intelligence in Sports, Capital University of Physical Education and Sports, Beijing, China

**Keywords:** endurance training, brain plasticity, gray matter, functional magnetic resonance imaging, functional reorganization

## Abstract

**Background:**

Although previous studies have shown that athletes engaged in endurance sports exhibit unique characteristics of brain plasticity, there has been no systematic investigation into the structural and functional brain characteristics of endurance athletes with varying training levels.

**Methods:**

Utilizing the “expert-novice paradigm” design, we employed functional magnetic resonance imaging (fMRI) to obtain images of brain structure and functional activity. We compared differences in gray matter volume (GMV), fractional amplitude of low-frequency fluctuations (fALFF), and degree centrality (DC) among high-level endurance athletes, moderate-level endurance athletes, and non-athlete controls.

**Results:**

(1) High-level endurance athletes exhibited significantly greater GMV in the left parahippocampal gyrus, bilateral thalamus, right temporal lobe, and bilateral cerebellum compared to both moderate-level endurance athletes and controls. The GMV in these regions showed an increasing trend with more years of endurance training and higher endurance capacity. Additionally, these athletes had significantly higher fALFF in the left superior medial frontal gyrus and right precuneus, as well as higher DC in the right lateral occipital lobe compared to moderate-level endurance athletes. They also had significantly higher DC in the right precuneus and cerebellum compared to the control group. (2) Moderate-level endurance athletes demonstrated significantly greater GMV in the right prefrontal cortex, bilateral medial frontal lobe, right temporal pole, right striatum, and bilateral insula compared to high-level endurance athletes. They also had significantly higher fALFF in the left posterior cingulate gyrus compared to high-level endurance athletes. (3) Control group showed significantly greater GMV in the right amygdala, higher fALFF in the left medial frontal lobe, and greater DC in the left lateral occipital lobe compared to moderate-level endurance athletes.

**Conclusion:**

Adaptive benefits exhibit different characteristics across different endurance levels. High-level endurance athletes exhibit pronounced enhancements in gray matter volume and functional activity in regions associated with memory, motor control, and sensory processing. While moderate-level athletes demonstrate distinct functional reorganization in the default mode network and cerebellum.

## 1 Background

Neuroplasticity refers to the brain’s adaptive adjustments in structure and function in response to experience or environmental changes, a phenomenon particularly pronounced in the field of sports. The neuroplasticity of brain structure and function in athletes is a key area of research in sports cognitive science. In high-level athletes, long-term training may lead to an increase in the volume or density of gray matter in brain regions involved in motor control. This change can be quantitatively assessed, providing valuable data to further explore the impact of sports on the brain. Studying the relationship between neuroplasticity and sports not only reveals the profound effects of physical activity on the brain but also helps us understand the neural mechanisms underlying motor skill acquisition, as well as the intrinsic impact of brain structure and functional plasticity on physical performance. Such understanding is crucial for enhancing training efficiency, optimizing training methods, and guiding practical athletic practices.

Multiple studies have shown that the neuroplasticity of athletes involved in endurance sports exhibits certain distinctive characteristics. For example, Wenzel et al. (2014) found that sprinters, jumpers, and throwers had greater gray matter volume in the rapid foot movement activation area of the anterior cerebellum compared to endurance athletes. This result preliminarily suggests that the neuroplasticity characteristics of endurance athletes differ from those of athletes in explosive sports. Following this, Schlaffke et al. (2014) compared the whole-brain differences among endurance athletes, martial artists, and control groups, finding that endurance athletes had greater gray matter volume in the left superior frontal gyrus and left parahippocampal gyrus compared to the control group, and greater gray matter volume in the dentate gyrus of the hippocampus compared to the martial arts experts. They further speculated that the reorganization of the medial frontal lobe and medial temporal lobe (hippocampus/parahippocampal gyrus) may be related to aerobic metabolism. Zhang et al. (2022) expanded this research with a larger sample size and stricter correction standards. The results showed that athletes in aerobic track and field events (10,000 meters, half marathon, 20 km race walk) had greater gray matter volume in the cerebellum and temporal lobe compared to athletes in anaerobic events (100, 200, 400 meters and 110-meter hurdles, long jump, triple jump, and javelin throw), but smaller gray matter volume in the basal ganglia compared to athletes in sprint and explosive events. Aerobic event athletes also had higher fALFF in motor areas of the frontal and parietal lobes and higher DC in the prefrontal cortex. Regarding brain networks, adolescents involved in high-level aerobic exercise exhibited differences in resting-state functional connectivity in motor control-related networks compared to sedentary individuals (Raichlen et al., 2016).

Although the aforementioned studies have confirmed the close relationship between endurance sports and neuroplasticity, no research has yet systematically explored the brain structure and functional characteristics of endurance athletes with varying training levels. Given that the training levels and years of experience among subjects differ, and that the acquisition of motor skills is a phased process, with different skill levels manifesting distinct central nervous system characteristics, it is necessary to conduct in-depth research on endurance athletes with different training levels. Therefore, this study employs the “expert-novice paradigm” to systematically examine the brain structure and functional activity characteristics of individuals with different endurance levels. Based on the findings and discussions from the existing literature, we propose the following hypotheses: (1) individuals with varying levels of endurance training will exhibit distinct differences in brain structure and functional characteristics; (2) these differences are likely to be localized in specific brain regions, including the frontal lobe, parietal lobe, cerebellum, hippocampus, and parahippocampal gyrus. This study aims to further understand the impact of sports on the brain, particularly among endurance athletes with varying training levels, and to provide a scientific basis for the guidance of sports training and skill development.

## 2 Materials and methods

### 2.1 Study design

This study was conducted as a cross-sectional investigation using a double-blind design. To minimize potential biases and preserve the integrity of the results, group assignments were concealed from all participants and researchers, with the exception of the first author.

### 2.2 Participants

A total of 76 participants were recruited for this study, comprising high-level endurance athletes (HG), moderate-level endurance athletes (MG), and a non-athlete control group (CG). The high-level endurance athletes (*n* = 26; 18 males, 8 females; mean age 21.1 ± 2.5 years) had extensive and systematic professional training experience (training duration ranging from 4 to 14 years, with an average of 7.5 ± 2.6 years). These athletes participated in high-level competitions or training and demonstrated significant athletic performance and expertise (all hold certification as China’s national first-level athletes or higher). Their training regimens were highly specialized, with both frequency and intensity being considerably high. The moderate-level endurance athletes (*n* = 27; 18 males, 9 females; mean age 22.1 ± 2.2 years) had shorter training durations (1 to 4 years, with an average of 2.3 ± 1.0 years) and lower training intensity compared to the high-level group. Although they lacked official athletic rankings, they frequently participated in public sports events, such as various marathon races. The control group (*n* = 23; 17 males, 6 females; mean age 21.0 ± 2.0 years) consisted of individuals who reported minimal participation in physical activities, engaging in sports less than once per week, aside from physical education classes, over the past year. The information on subjects’ age, height, weight, and BMI is shown in Table 1.

**Table 1:** Basic information of subjects.

	HG (*n* = 26)	MG (*n* = 27)	CG (*n* = 23)	*F*	*p*
Age (year)	21.1 ± 2.5	22.1 ± 2.2	21.0 ± 2.0	2.018	0.140
Height (cm)	175.1 ± 8.9	172 ± 6.6	171.5 ± 7.0	1.709	0.188
Weight (kg)	64.4 ± 9.0	65.1 ± 6.8	66.3 ± 12.8	0.255	0.776
BMI (kg/cm^2^)	20.88 ± 1.25	21.97 ± 1.5	22.4 ± 3.13^a^	3.570	0.033

a Significant difference compared with the high-level group (*p* < 0.05).

All participants were right-handed, had no history of neurological disorders, and met the requirements for magnetic resonance imaging (MRI) scans. Informed consent was obtained from all participants prior to the experiment. This study was approved by the Ethics Committee of the School of Medicine at Tsinghua University (Ethics Approval Number: 20180016).

### 2.3 Image acquisition

This study employed a Philips Achieva 3.0 T MRI scanner with a 32-channel head coil to acquire brain imaging data from participants. T1-weighted imaging sequences were used to capture structural signals of the brain’s gray matter, with the following parameters: flip angle = 8°, number of slices = 180, slice thickness = 1 mm, acquisition matrix = 80 × 80, and field of view (FOV) = 230 × 230 mm. Additionally, a single-shot echo planar imaging sequence was utilized to obtain blood oxygen level dependent (BOLD) signals, with the following parameters: TR = 2 s, TE = 30 ms, flip angle = 90°, number of slices = 37, slice thickness = 3 mm, acquisition matrix = 80 × 80, FOV = 230 × 230 mm, and voxel size = 2.87 × 2.87 × 3.50 mm^3^. Participants were instructed to “close their eyes, relax, stay awake, and avoid thinking about specific things” during the BOLD scan. To minimize the auditory and psychological impact of noise from gradient field transitions, participants wore earplugs and noise-canceling headphones. Additionally, to reduce the effects of head movement on image quality, participants were advised to remain as still as possible during the scan, and foam padding was used to fill the gaps between their heads and the coil.

### 2.4 Data preprocessing

Image preprocessing was conducted using the RESTplus software package (Jia et al., 2019) and involved several steps to ensure data quality and accuracy. Initially, the data from the first five time points were deleted to remove any initial transients. Next, time correction was applied by performing interpolation with the middle slice (Slice 37) serving as the reference layer. Head motion correction followed, where data exhibiting translations greater than 2 mm or rotations exceeding 2° were excluded, with all participants successfully meeting this criterion. For spatial normalization, brain images were segmented into gray matter, white matter, and cerebrospinal fluid, and each tissue type was mapped to the standard MNI template to facilitate comparative analysis. The self-T1 structural image was used for registration in this study. Gaussian smoothing was then performed to enhance the signal-to-noise ratio of fMRI images and ensure the data approximated a Gaussian random field, utilizing a full width at half maximum (FWHM) smoothing kernel of 8 × 8 × 8 mm^3^. Finally, covariates such as whole brain white matter and cerebrospinal fluid signals, as well as 24 Friston head motion parameters, were removed to account for unwanted variations. The quality of the images was meticulously checked and controlled at each preprocessing step.

### 2.5 Data analysis

#### 2.5.1 Voxel-based morphometry analysis

Voxel-based morphometry (VBM) analysis (Ashburner and Friston, 2000; Mechelli et al., 2005) was conducted using the VBM8 software package.^1^ The analysis process commenced with the removal of noise signals from the skull, scalp, and other non-brain tissues to reduce artifacts. High-resolution T1-weighted images were then subjected to normalization using the DARTEL (Diffeomorphic Anatomical Registration Through Exponentiated Lie Algebra) method, which aligns images to a standard space for consistent comparison across subjects. Subsequent to normalization, the images were segmented into different tissue types, including gray matter, white matter, and cerebrospinal fluid. This segmentation process is crucial for accurately quantifying brain structures and was followed by the registration of these tissue segments to the MNI (Montreal Neurological Institute) template to facilitate inter-subject comparison. Finally, the images were smoothed with an 8 × 8 × 8 mm^3^ full width at half maximum (FWHM) smoothing kernel, a step that improves the signal-to-noise ratio and enhances the statistical power by mitigating the effects of anatomical variability. The outcome of these preprocessing steps is the generation of whole-brain gray matter volume (GMV) distribution maps, which are essential for assessing structural brain differences and changes across subjects or conditions.

The amplitude of low-frequency fluctuations (ALFF) is a frequency domain analysis method used to quantify the total power of brain activity within the low-frequency range (0.01–0.1 Hz) (Greicius et al., 2009). This measure reflects the intensity of spontaneous brain activity. To enhance the representation of low-frequency signals, ALFF is normalized by the total energy across the entire frequency spectrum to yield fractional ALFF (fALFF) (Zou et al., 2008). This normalization is predicated on the premise that low-frequency fluctuations are indicative of intrinsic neural activity and functional connectivity, providing valuable insights into brain function and various neurological and psychiatric conditions. Prior to calculating ALFF and fALFF, rigorous preprocessing steps—including motion correction, spatial normalization, and temporal filtering—are performed to mitigate artifacts and ensure data accuracy. Subsequent statistical analyses of fALFF are conducted to examine differences between conditions or correlate fALFF values with behavioral and clinical measures. Elevated fALFF values in specific brain regions are interpreted as indicative of increased spontaneous neural activity, whereas reduced values may reflect diminished activity. In this study, the RESTplus software package was utilized to compute the whole-brain fALFF distribution map following Fisher’s *z*-transform, which enhances statistical reliability by stabilizing variance.

**Figure 1:**
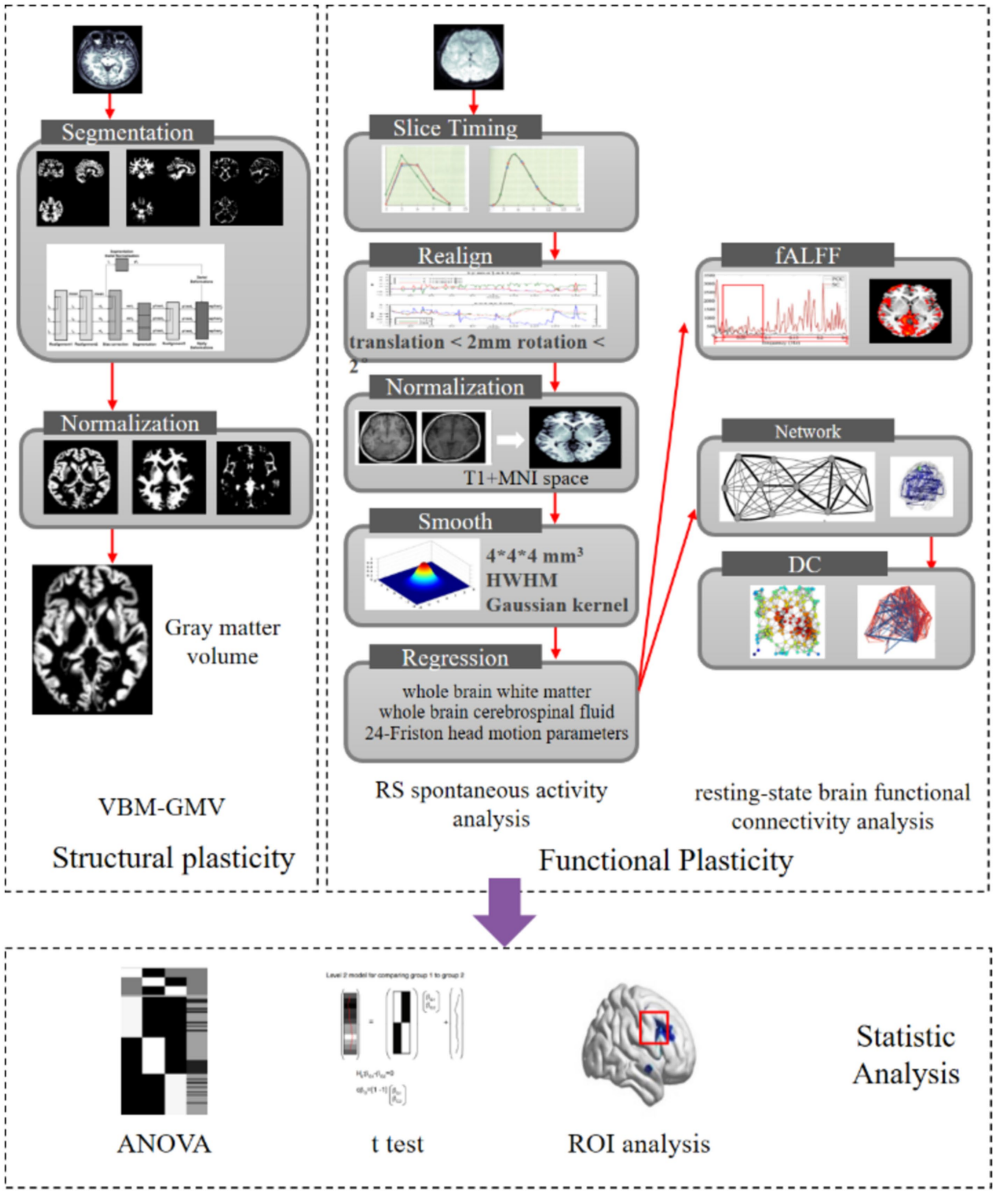
fMRI scanning and image processing flowchart.

Degree centrality (DC) is a metric derived from graph theory used to assess the prominence or influence of individual brain regions within the global brain network (Bullmore and Sporns, 2009). A higher DC value signifies greater connectivity and centrality of a brain region, highlighting its critical role in facilitating network-wide communication. In this study, the RESTplus software package was employed to threshold the inter-node connections at a correlation coefficient of *r* = 0.25, resulting in an undirected weighted matrix that represents the complex brain network. Subsequently, the whole-brain DC distribution map was computed following Fisher’s *z*-transformation, which normalizes the correlation coefficients and enhances statistical reliability by stabilizing variance. This methodological approach enables a robust evaluation of the centrality of brain regions and their contributions to the overall network connectivity. For the process of fMRI scanning and image processing, see Figure 1.

### 2.6 Statistical analysis

Statistical analyses of whole-brain gray matter volume (GMV), fractional ALFF (fALFF), and degree centrality (DC) data were performed using the SPM12 software package. GMV was analyzed to evaluate structural plasticity, while fALFF and DC were assessed to investigate functional plasticity. Referring to paradigms used in previous studies (Yang et al., 2022; Zhao et al., 2023), we first applied a one-way analysis of covariance (ANCOVA), a parametric analysis, to compare differences in whole-brain gray matter volume (GMV), fractional ALFF (fALFF), and degree centrality (DC) across the three subject groups. Independent-sample *t*-tests were then conducted to examine pairwise group differences. Age was included as a covariate in the statistical calculations. A significance threshold of *p <* 0.01 was applied. Given that statistical tests were conducted independently for each voxel, which constitutes multiple comparisons, the risk of type I errors (false positives) is markedly increased when numerous voxels are tested simultaneously, potentially leading to erroneous results. To address this concern and reduce the incidence of false positives, Gaussian random field (GRF) theory-based multiple comparison correction was applied to the fMRI data. This method adjusts the statistical threshold to control error probabilities across multiple voxel-level tests throughout the brain. For regions demonstrating significant differences, further voxel-wise family-wise error (FWE) correction was applied using the Bonferroni method, providing a stringent adjustment for multiple comparisons. This approach aims to minimize both type I errors, thereby ensuring a robust and scientifically valid outcome. The corrected results were presented in tabular form and visualized in the MNI standard space using the RESTplus and BrainNet View software packages. ROI-based analyses were subsequently performed. Regions of interest (ROIs) were defined with a 6 mm radius centered on the peak voxel coordinates of significant differences identified in the previous ANOVA and *t*-test results. GMV, fALFF, and DC values for each subject were extracted from these ROIs. A one-way analysis of variance was then conducted on these extracted values using SPSS, followed by *post hoc* LSD tests, to further examine the trends in plasticity characteristics across the three groups. Similarly, age was included as a covariate in the statistical calculations.

## 3 Results

### 3.1 Overall analysis of brain structural and functional variance

**Figure 2:**
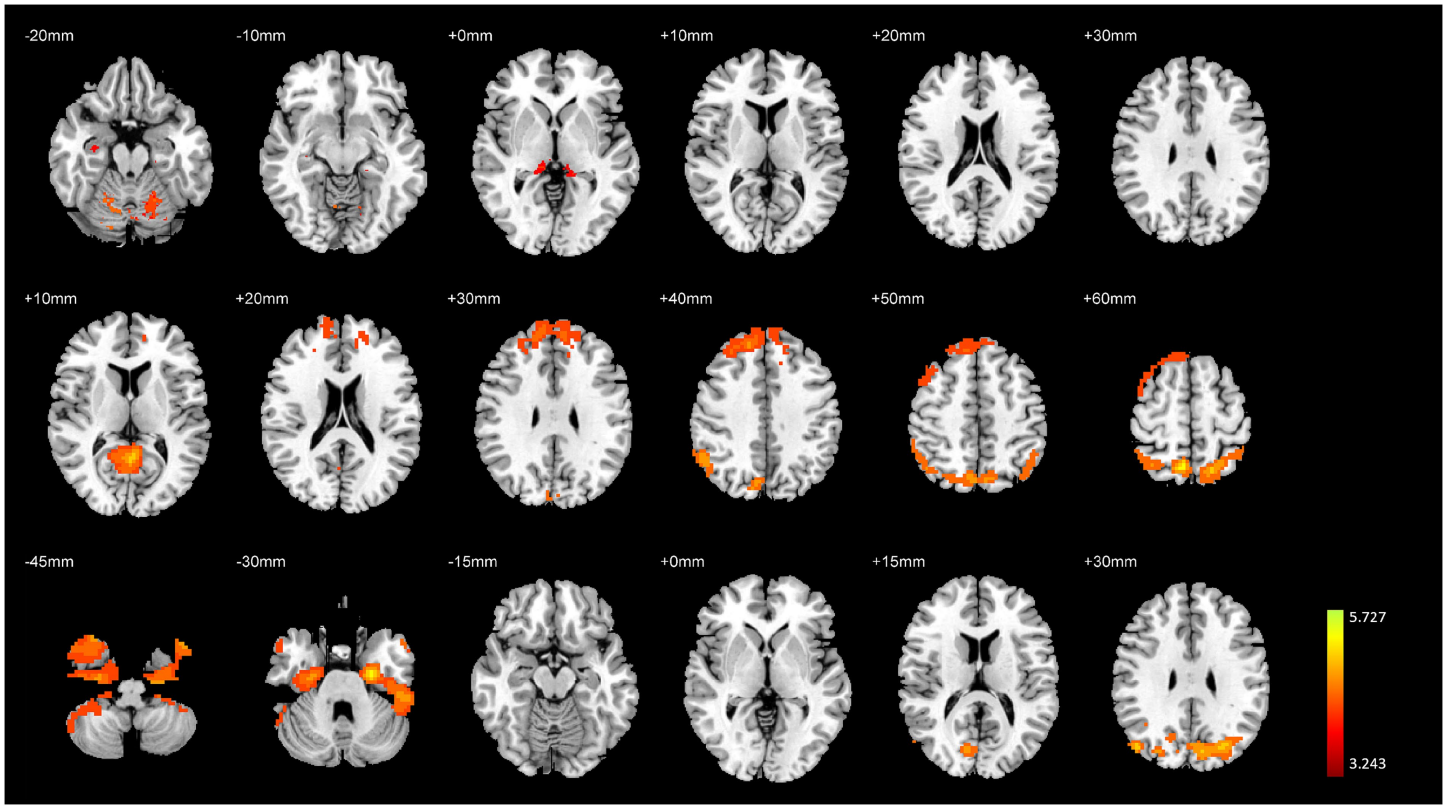
Variance analysis results of brain structural and functional differences among the three groups. The top to bottom rows represent the results for GMV, fALFF, and DC, respectively.

The overall analysis results of brain structural and functional variability are presented in Table 2 and Figure 2. The analysis of variance indicated significant differences among the three groups of subjects concerning brain structure (GMV) and brain function (fALFF and DC) (*p <* 0.01, FWE corrected). Specifically, significant variations in GMV were observed in the bilateral hippocampus, bilateral parahippocampal gyrus, and bilateral cerebellum (*p <* 0.01, FWE corrected). For fALFF, significant differences were found in the right medial superior frontal gyrus, right inferior temporal gyrus, right precuneus, and left cerebellar vermis (*p <* 0.01, FWE corrected). Additionally, significant differences in degree centrality (DC) were noted in the left entorhinal cortex, right angular gyrus, and right cerebellum (*p <* 0.01, FWE corrected).

**Table 2:** Variance analysis results.

Location	Hemisphere	Cluster FWE *p*	Cluster size	Coordinate *x*	Coordinate *y*	Coordinate *z*	Peak *T*
GMV							
Hippocampus	L	0.002	86	−12	−36	3	3.735
Hippocampus	R	<0.001	107	32	−18	−23	3.243
Parahippocampal gyrus	R	<0.001	198	18	2	−35	4.660
Parahippocampal gyrus	R	<0.001	134	11	−32	−2	4.214
Parahippocampal gyrus	L	0.001	90	−3	−84	−2	4.697
Cerebellum apex	L	<0.001	459	−27	−68	−24	4.252
Cerebellum apex	R	<0.001	155	18	−63	−23	3.409
Cerebellum basal	R	0.009	71	18	−51	−60	3.363
Cerebellum posterior	R	<0.001	590	36	−78	−32	4.264
Cerebellum posterior	L	0.007	74	−2	−74	−23	3.678
Cerebellum posterior superior	L	<0.001	135	−35	−78	−27	3.973
Cerebellum posterior middle	R	<0.001	132	24	−83	−36	4.028
Cerebellum posterior inferior	R	<0.001	399	17	−69	−50	4.369
Cerebellum posterior inferior	L	<0.001	407	−11	−74	−45	4.208
fALFF							
Medial superior frontal gyrus	R	<0.001	938	15	42	39	4.188
Inferior temporal gyrus	R	0.042	177	69	−27	−21	3.826
Superior parietal lobule-precuneus	R	<0.001	3,406	6	−60	63	5.727
Top of cerebellar vermis	L	<0.001	1,322	−6	−51	3	5.286
DC							
Hippocampus, entorhinal cortex	L	<0.001	1,139	−24	−15	−33	4.891
Superior parietal lobule-angular gyrus	R	<0.001	5,825	39	−57	36	4.874
Cerebellum	R	0.009	636	27	−18	−33	3.816

FWE corrected, *p* < 0.01, activation coordinates are reported in MNI space.

### 3.2 Comparison of brain structure and function between HG and MG

**Figure 3:**
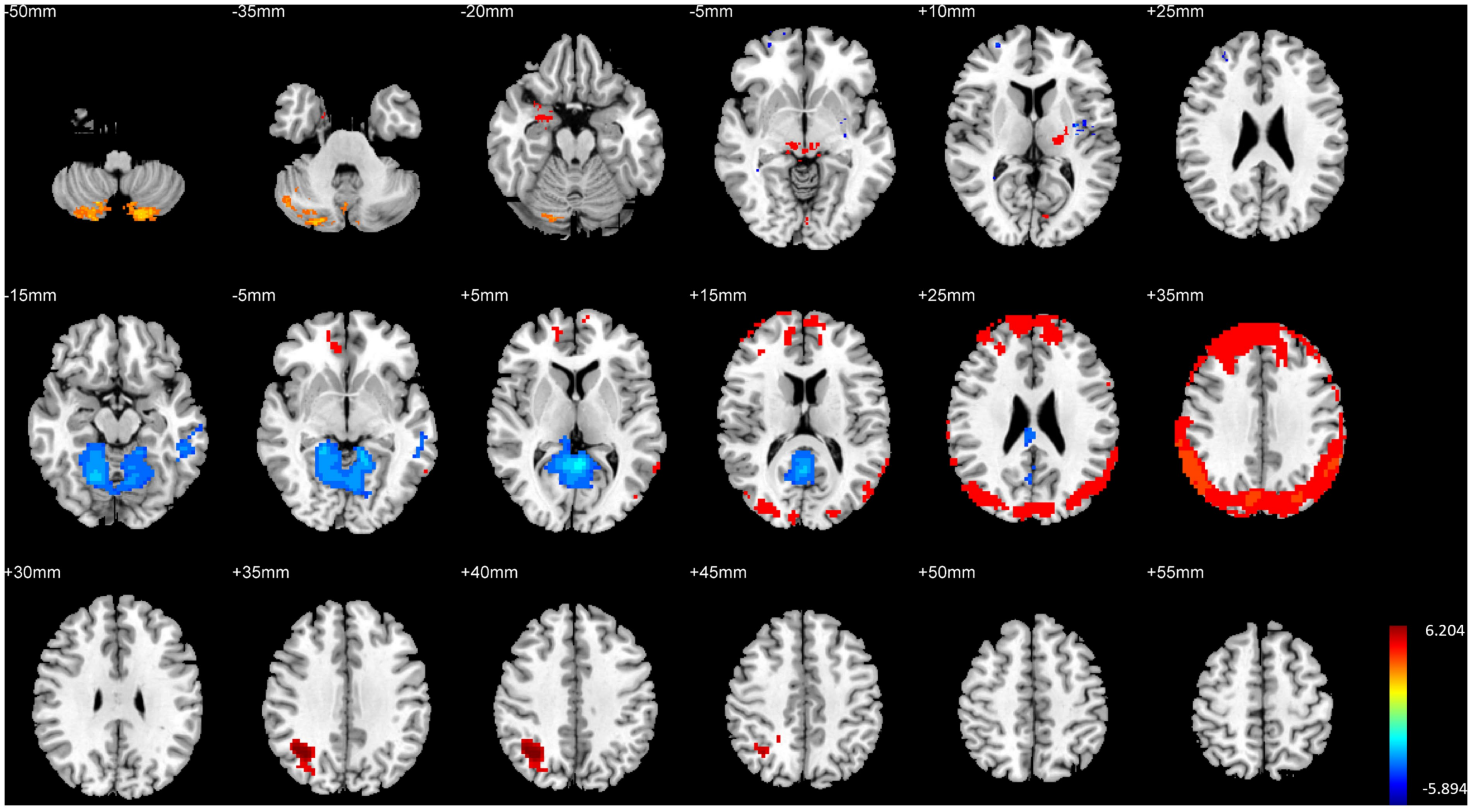
Illustration of brain structural and functional differences between HG and MG. The rows from top to bottom correspond to results for GMV, fALFF, and DC, respectively.

HG exhibited significantly higher GMV in the left parahippocampal gyrus, bilateral thalamus, right temporal lobe, and bilateral cerebellum compared to MG. Additionally, fALFF in the left medial superior frontal gyrus and right precuneus was significantly higher in HG than in MG. DC in the right lateral occipital lobe was also significantly higher in HG than in MG (*p <* 0.01, FWE corrected, see Table 3 and Figure 3).

**Table 3:** Brain regions exhibiting structural and functional differences between HG and MG.

Location	Hemisphere	Cluster FWE *p*	Cluster size	Coordinate *x*	Coordinate *y*	Coordinate *z*	Peak *T*
GMV							
Parahippocampal gyrus	L	0.002	131	−3	−84	−2	4.885
Parahippocampal gyrus	L	<0.001	215	−5	−26	−5	4.095
Thalamus	R	<0.001	327	12	−30	−2	4.428
Thalamus	L	0.001	138	−18	−23	12	4.984
Temporal lobe	R	<0.001	360	29	5	−18	4.313
Posterior cerebellar lobe superior	L	<0.001	1,493	−35	−78	−27	4.831
Posterior cerebellar lobe middle	R	<0.001	2,592	23	−83	−36	4.921
Posterior cerebellar lobe inferior	L	<0.001	1,031	−17	−77	−51	4.700
Prefrontal lobe	R	0.015	103	29	−1	45	−4.904
Medial frontal lobe	R	0.004	122	33	45	17	−4.093
Medial frontal lobe	R	0.005	119	26	54	6	−3.709
Medial frontal lobe	L	0.001	136	−11	24	60	−3.671
Temporal pole	R	0.001	146	18	−102	−2	−4.320
Striatum	R	0.015	103	33	−40.5	6	−3.558
Insula	R	0.003	124	30	14	0	−3.639
Insula	R	<0.001	159	35	21	−11	−4.399
Insula	L	<0.001	229	−33	−9	14	−4.613
fALFF							
Medial superior frontal gyrus	L	<0.001	2,031	−3	51	45	4.884
Superior parietal lobule-precuneus	R	<0.001	4,961	6	−60	63	6.204
Posterior cingulate gyrus	L	<0.001	5,346	−3	−51	6	−5.894
DC							
Lateral occipital lobe	R	<0.001	25,754	33	−63	39	6.081

FWE corrected, *p* < 0.01, activation coordinates are reported in MNI space. A positive *T*-value at the peak indicates that HG is greater than MG, while a negative *T*-value indicates that HG is less than MG.

Conversely, MG showed significantly higher GMV in the right prefrontal cortex, bilateral medial frontal cortex, right temporal pole, right striatum, and bilateral insula compared to HG. Furthermore, fALFF in the posterior cingulate gyrus was significantly higher in MG than in HG (*p <* 0.01, FWE corrected, see Table 3 and Figure 3).

### 3.3 Comparison of brain structure and function between MG and control

**Figure 4:**
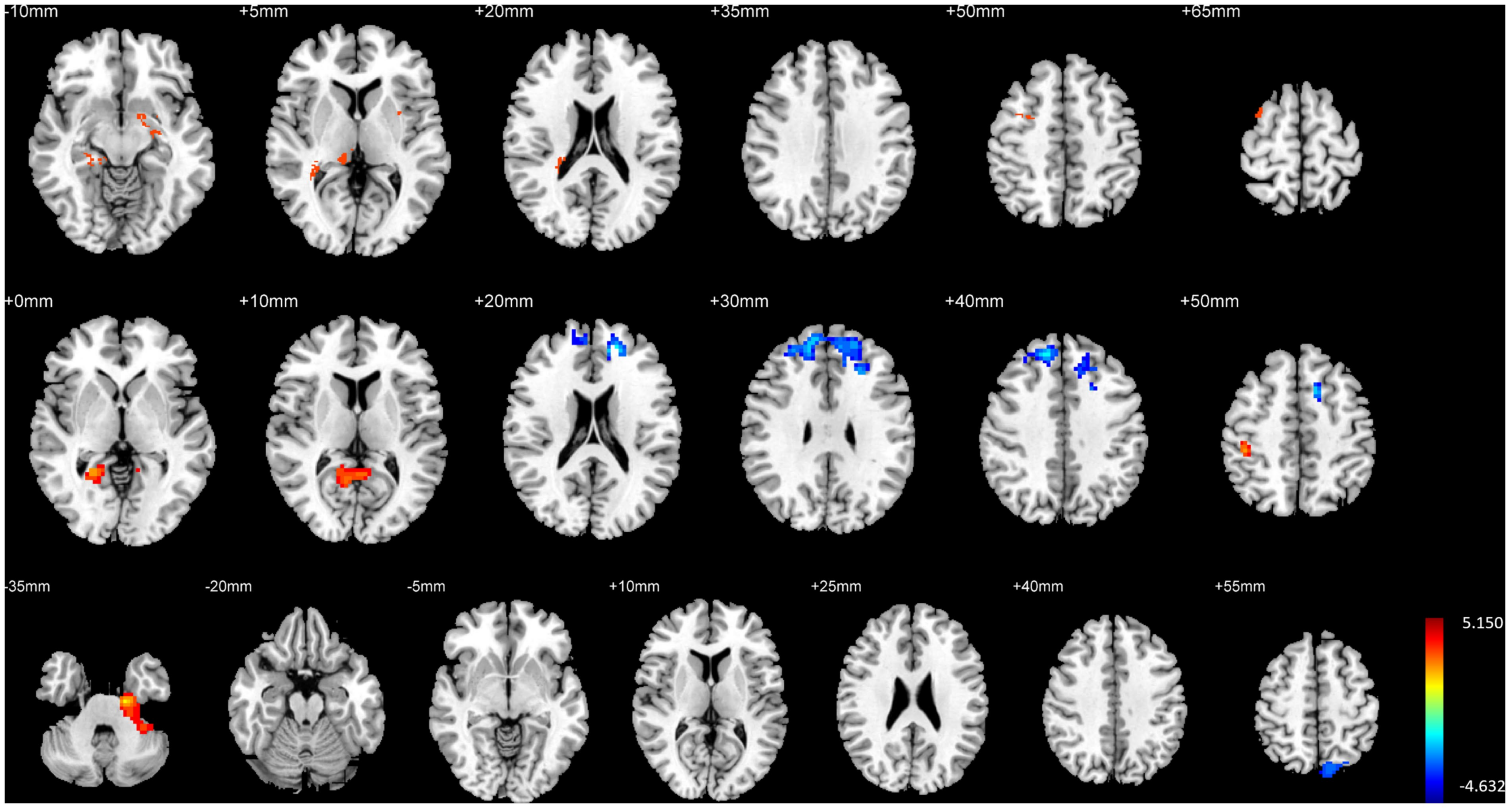
Illustration of brain structural and functional differences between MG and CG. The rows from top to bottom correspond to results for GMV, fALFF, and DC, respectively.

MG exhibited significantly higher GMV in the right prefrontal cortex, right precuneus, left parahippocampal gyrus, right putamen, and right insula compared to CG. Additionally, fALFF in the right prefrontal cortex and right parahippocampal gyrus was significantly higher in MG than in CG, and DC in the left cerebellum was also significantly higher in MG (*p <* 0.01, FWE corrected, see Table 4 and Figure 4).

**Table 4:** Brain regions exhibiting structural and functional differences between MG and CG.

Location	Hemisphere	Cluster FWE *p*	Cluster size	Coordinate *x*	Coordinate *y*	Coordinate *z*	Peak *T*
GMV							
Prefrontal lobe	R	0.001	139	41	0	60	3.655
Precuneus	R	0.012	107	17	−63	63	3.780
Parahippocampal gyrus	L	0.036	92	−29	−4	−17	4.090
Putamen	R	0.049	88	35	−42	4	4.097
Insula	R	0.021	99	35	15	−2	3.759
Amygdala	R	0.024	97	9	−32	0	−3.678
fALFF							
Lateral frontal lobe	R	0.012	332	12	3	69	3.724
Parahippocampal gyrus	R	0.039	262	24	−51	3	3.686
Medial frontal lobe	L	<0.001	580	−18	48	21	−3.825
DC							
Cerebellum	L	0.020	1,319	−18	−18	−33	5.150
Lateral occipital lobe	L	<0.001	8,708	−15	−66	66	−4.632

FWE corrected, *p* < 0.01, activation coordinates are reported in MNI space. A positive *T*-value at the peak indicates that MG is greater than CG, while a negative *T*-value indicates that MG is less than CG.

Conversely, CG showed significantly higher GMV in the right amygdala compared to MG. fALFF in the left medial frontal cortex was significantly higher in CG than in MG, and DC in the left lateral occipital lobe was significantly higher in CG than in MG (*p <* 0.01, FWE corrected, see Table 4 and Figure 4).

### 3.4 Comparison of brain structure and function between HG and control

**Figure 5:**
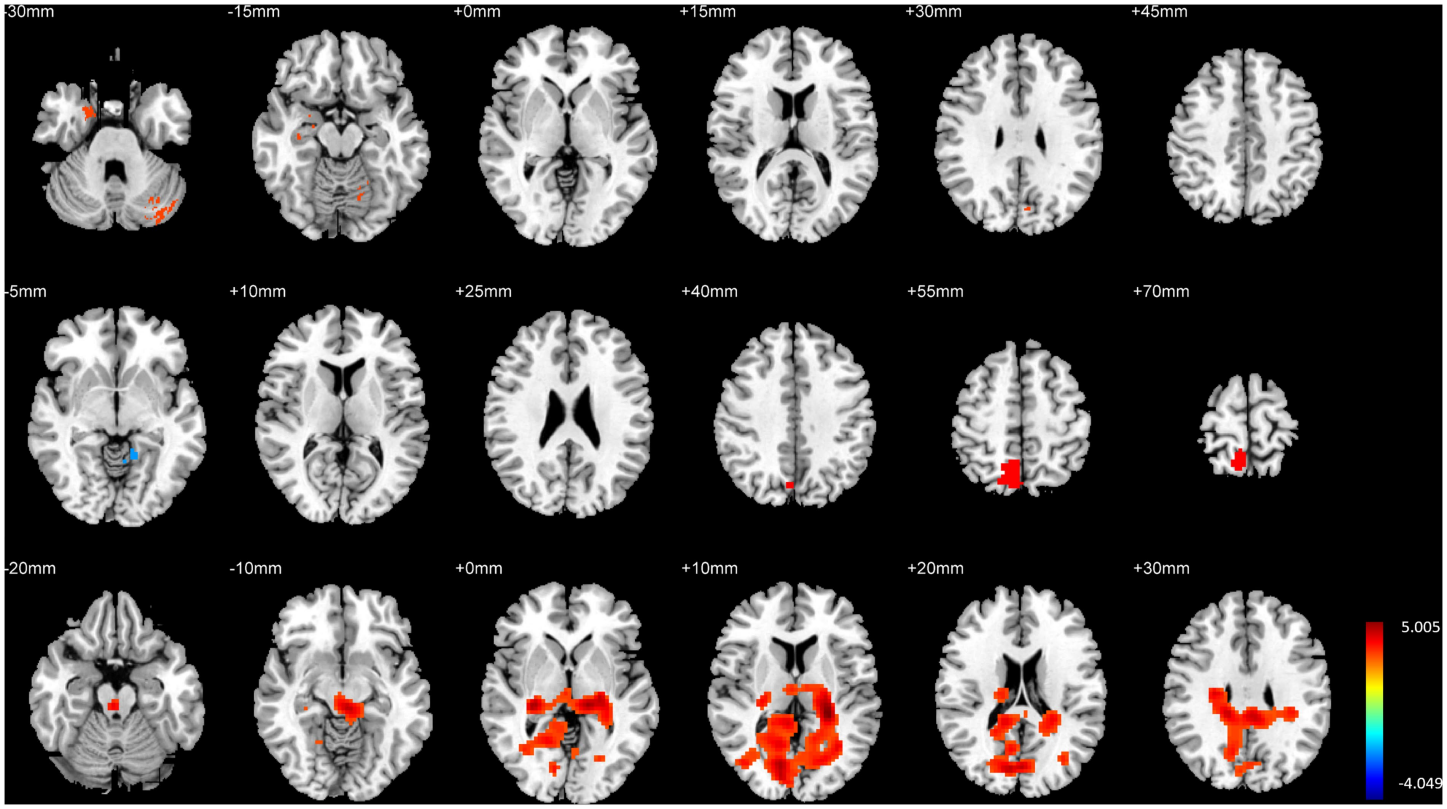
Illustration of brain structural and functional differences between HG and CG. The rows from top to bottom correspond to results for GMV, fALFF, and DC, respectively.

HG exhibited significantly higher GMV in the right hippocampal entorhinal cortex, left parahippocampal gyrus, right precuneus, and bilateral cerebellum compared to CG. Additionally, fALFF in the right precuneus was significantly higher in HG than in the control group, and DC in the left parahippocampal gyrus and right hippocampus/parahippocampal gyrus was also significantly higher in HG compared to the control group (*p <* 0.01, FWE corrected, see Table 5 and Figure 5).

**Table 5:** Brain regions exhibiting structural and functional differences between HG and CG.

Location	Hemisphere	Cluster FWE *p*	Cluster size	Coordinate *x*	Coordinate *y*	Coordinate *z*	Peak *T*
GMV							
Hippocampus, entorhinal cortex	R	<0.001	716	18	2	−35	4.842
Parahippocampal gyrus	L	<0.001	189	−20	−12	−29	3.781
Precuneus	L	0.013	106	−8	−81	41	3.825
Cerebellum	L	<0.001	2,655	−26	−69	−24	4.664
Cerebellum	R	0.008	112	8	−66	−36	3.630
Cerebellum	R	<0.001	259	17	−51	−62	3.731
fALFF							
Precuneus	R	<0.001	3,400	9	−63	60	5.005
Inferior temporal gyrus	R	0.007	357	69	−27	−21	−4.049
Inferior temporal gyrus	L	0.001	520	−15	−48	−6	−3.866
DC							
Parahippocampal gyrus	L	0.002	2,231	−27	−33	9	3.606
Hippocampus/Parahippocampal gyrus	R			27	−27	3	3.432

FWE corrected, *p* < 0.01, activation coordinates are reported in MNI space. A positive *T*-value at the peak indicates that HG is greater than CG, while a negative *T*-value indicates that HG is less than CG.

Conversely, CG demonstrated significantly higher fALFF in the bilateral inferior temporal gyri compared to HG (*p <* 0.01, FWE corrected, see Table 5 and Figure 5).

### 3.5 ROI analysis results

**Figure 6:**
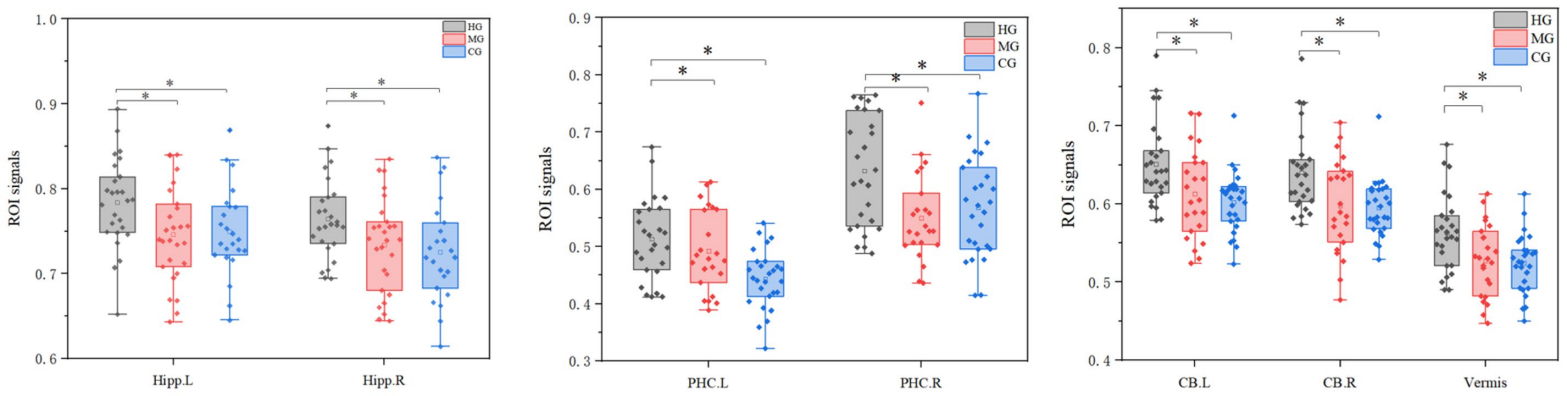
ROI analysis results for structural plasticity. Hipp, hippocampus; PHC, parahippocampal gyrus; CB, cerebellar; CB vermis, cerebellar vermis; R, right; L, left. The vertical axis represents the signal value of GMV within the ROI. ^*^p < 0.05 and ^**^p < 0.01.

The ROI analysis results for structural plasticity are shown in Figure 6. The effect sizes (partial *η*^2^) were as follows: left hippocampus (*η*^2^*p* = 0.14), right hippocampus (*η*^2^*p* = 0.15), left cerebellum (*η*^2^*p* = 0.20), right cerebellum (*η*^2^*p* = 0.18), vermis (*η*^2^*p* = 0.20), left posterior hippocampal cortex (*η*^2^*p* = 0.23), and right posterior hippocampal cortex (*η*^2^*p* = 0.15), indicating notable structural differences between groups. GMV of the hippocampus, parahippocampal gyrus, and cerebellum in the HG was significantly higher than in the other two groups. Furthermore, the GMV of these regions exhibited an overall increasing trend with longer endurance training duration and improved endurance capacity.

**Figure 7:**
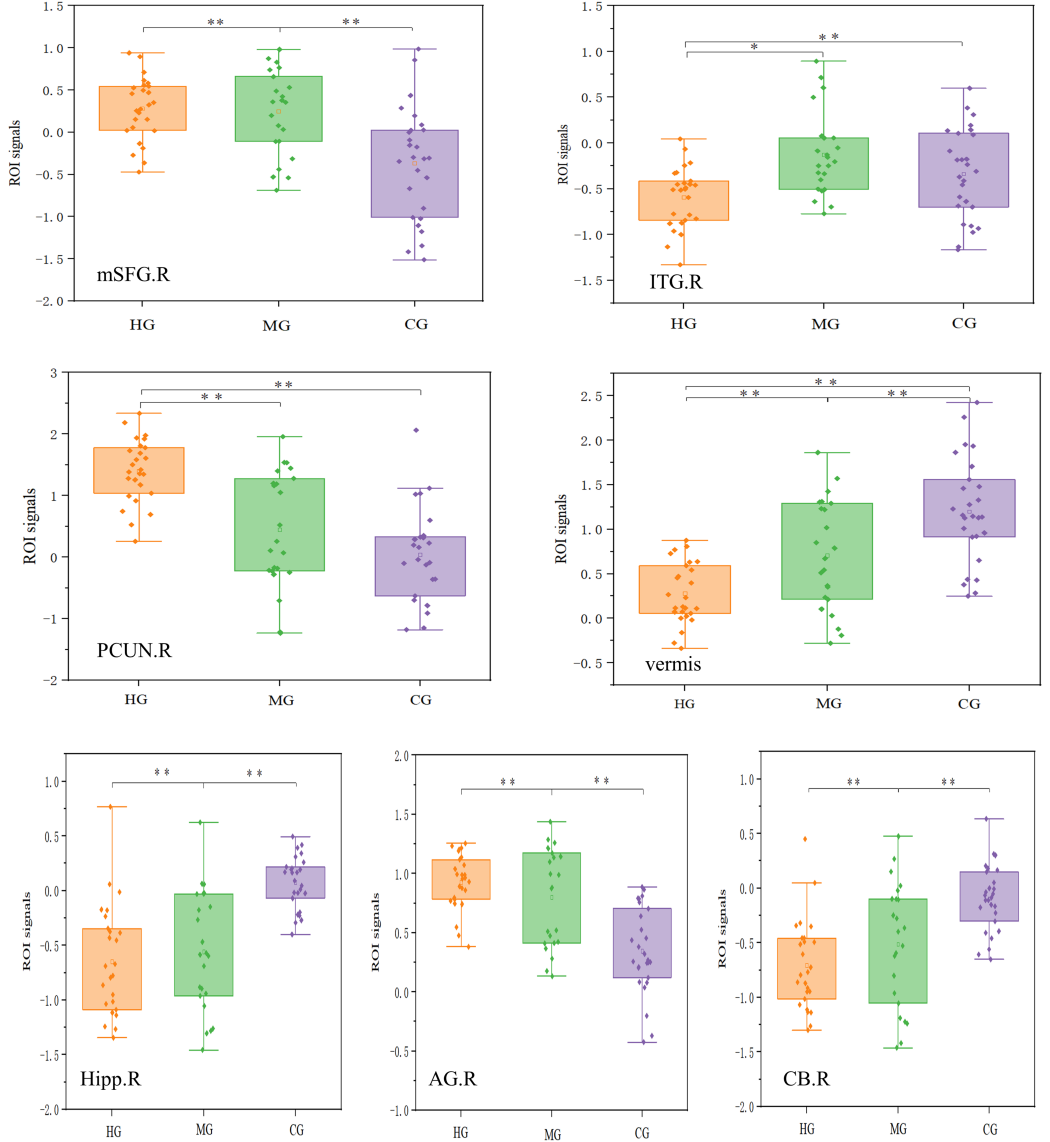
ROI analysis results for functional plasticity. mSFG = medial superior frontal gyrus, ITG = inferior temporal gyrus, CB = cerebellum, PCUN = precuneus, vermis = Cerebellar vermis, Hipp = Hippocampus, AG =angular gyrus. R = right, L = left. The top row of four images represents the fALFF results, while the bottom row of three images represents the DC results. The vertical axis in each case represents the signal value of the corresponding metric within the ROI. ^*^p < 0.05, ^**^p < 0.01.

The effect sizes of fALFF were as follows: right CUN (*η*^2^*p* = 0.40), vermis (*η*^2^*p* = 0.36), right ITG (*η*^2^*p* = 0.22), and right MFG (*η*^2^*p* = 0.27). While the effect sizes of DC were as follows: right hippocampus (*η*^2^*p* = 0.39), right ANG (*η*^2^*p* = 0.39), and right cerebellum (*η*^2^*p* = 0.35). The ROI analysis results for functional plasticity are illustrated in Figure 7. The fALFF in the medial superior frontal gyrus and DC in the cerebellum showed an initial increase followed by a decrease with longer endurance training duration and enhanced endurance capacity, with the MG significantly higher than the other two groups. Conversely, fALFF in the precuneus and cerebellar vermis, as well as DC in the hippocampus and angular gyrus, demonstrated an initial decrease followed by an increase with increased training duration and improved endurance capacity, with MG significantly lower than the other two groups.

## 4 Discussion

This study aims to systematically investigate the characteristics of brain structure and functional activity across individuals with varying levels of endurance. The study involves three distinct groups of subjects, each representing different levels of endurance attributable to differences in training duration, intensity, and expertise. By comparing brain structure and functional plasticity across these groups, we seek to elucidate the relationship between endurance levels and corresponding neural characteristics.

### 4.1 Reorganization in regions associated with memory, emotion, and motor control in HG

The hippocampus is related to memory and emotion (Gorham et al., 2019; Maguire et al., 2000; Miller and Hen, 2015). Previous studies have demonstrated that endurance athletes exhibit increased gray matter volume in the left parahippocampal gyrus compared to a control group of non-athletes, and show greater gray matter volume in the dentate gyrus of the hippocampus compared to a group of martial artists who are also athletes (Schlaffke et al., 2014). The hippocampal regions are closely associated with learning and memory, and increased aerobic endurance is linked to plasticity changes in the hippocampus (Burdette et al., 2010; Chaddock et al., 2010). Burdette et al. (2010) found that following a four-month exercise intervention, elderly individuals in the exercise group exhibited increased cerebral blood flow (CBF) in the hippocampal regions and enhanced functional connectivity between the hippocampus and the anterior cingulate cortex. Erickson et al. (2009) observed that in elderly individuals, higher levels of aerobic endurance were significantly associated with increased hippocampal volume, indicating that greater endurance capacity is related to better memory function. Chaddock et al. (2010) also found that children with higher endurance capacity exhibited larger bilateral hippocampal volumes and better performance on memory tasks. Furthermore, bilateral hippocampal volume was found to mediate the relationship between maximal oxygen uptake and memory task performance. In this study, differences in gray matter volume among high-level endurance athletes, moderate-level endurance athletes, and sedentary controls were predominantly observed in the bilateral hippocampus and parahippocampal gyrus. These findings are consistent with previous preliminary research exploring brain structure and functional activity in endurance athletes (Schlaffke et al., 2014; Zhang et al., 2022). The hippocampus’s sensitivity to exercise-related plasticity may be associated with neurogenesis. Research indicates that physical activity can enhance a range of neurogenic processes in the hippocampal dentate gyrus, including the proliferation of neural stem cells and progenitor cells, thereby promoting neurogenesis. Consequently, the increased hippocampal gray matter volume observed in high-level endurance athletes is indicative of enhanced memory and emotional function.

In previous research conducted by our group, a comparison between aerobic and anaerobic athletes revealed that aerobic athletes exhibited greater cerebellar gray matter volume compared to their anaerobic counterparts (Zhang et al., 2022). The results of this study further confirm that endurance athletes have a larger cerebellar volume. The cerebellum, situated posteriorly and inferiorly to the cerebral hemispheres and connected to the brainstem, consists of several distinct regions. Its surface is covered by the cerebellar cortex, which is divided into two hemispheres. The underlying white matter contains the deep cerebellar nuclei, including the red nucleus and the oculomotor nucleus. The cerebellum is intimately involved in motor function, receiving sensory inputs from the spinal cord as well as from cortical and subcortical areas. It integrates these inputs to precisely regulate and fine-tune motor activities (Fine et al., 2002). The cerebellum is critical for motor timing and execution (Mauk et al., 2000), it facilitates the prediction of sensory outcomes associated with motor actions and detects discrepancies between predicted and actual sensory feedback (Hardwick et al., 2013; Penhune and Steele, 2012). Additionally, the cerebellum is involved in controlling motor speech, oculomotor functions, grip strength, voluntary limb movements, and classical conditioning reflexes (Manto et al., 2012). Previous research has indicated that prolonged motor skill training leads to plastic changes in cerebellar function. For instance, professional badminton players exhibit significantly higher gray matter density in the right cerebellum compared to the general population (Di et al., 2012). World-class mountaineers have been found to have significantly larger volumes of the cerebellar vermis lobules I–V compared to control groups (Di Paola et al., 2013). Similarly, basketball players exhibit increased cerebellar volumes relative to the general population (Park et al., 2009), while gymnastics training has been shown to induce significant alterations in cerebellar network parameters (Huang et al., 2018). Furthermore, short-term motor skill training can induce plastic changes in the cerebellum. Increased cerebellar volume is indicative of superior motor integration, modulation, and control capabilities in high-level endurance athletes.

Detailed pairwise comparisons and region-of-interest (ROI) analyses revealed that high-level endurance athletes exhibited significantly greater gray matter volumes in the bilateral hippocampus, left parahippocampal gyrus, bilateral cerebellum, and cerebellar vermis compared to the other two groups. These findings suggest that the gray matter volumes in the hippocampus, parahippocampal gyrus, and cerebellum generally increase with both the duration of endurance training and the level of endurance capacity.

### 4.2 Reorganization in the default network and cerebellum in MG

The default mode network (DMN) primarily consists of the medial prefrontal cortex and posterior parietal cortex (Martinelli et al., 2013), and research on the DMN has played a pivotal role in the study of resting-state brain activity. Shulman et al. (1997) first observed that certain brain regions show increased activation during rest, unrelated to specific tasks. Subsequently, Raichle et al. (2001) confirmed the existence of these regions using PET imaging and termed it the “default mode of brain function.” Although research on the DMN is still in its early stages and its functional mechanisms are not yet fully understood, significant progress has been made in clinical applications, particularly in the context of Alzheimer’s disease, depression, and their treatment (Jones et al., 2011; Koch et al., 2012; Marchetti et al., 2012; Mevel et al., 2011; Sheline et al., 2009).

In the field of sports science research, functional reorganization of the default mode network (DMN) has been identified in elite athletes, including world-class gymnasts (Wang et al., 2013), national-level basketball players (Tan et al., 2017), and long-distance runners (Raichlen et al., 2016). The findings of this study indicate that functional reorganization also takes place within the default mode network regions of long-distance runners. To date, no studies have directly compared resting-state fALFF levels between endurance athletes and non-athletes. However, our research group’s prior comparative analysis of aerobic and anaerobic athletes revealed that aerobic athletes exhibited higher fALFF in the motor regions of the frontal and parietal lobes compared to their anaerobic counterparts. This suggests that aerobic athletes have greater resting-state spontaneous activity in the frontal and parietal lobes (Zhang et al., 2022). The findings of this study further revealed that the differences in fALFF among high-level endurance athletes, average-level endurance athletes, and non-athlete controls were more extensively distributed across brain regions, including the right medial superior frontal gyrus, right inferior temporal gyrus, right parietal precuneus, and left cerebellar vermis. The precuneus is part of the parietal lobe and is involved in integrating somatosensory and visual information and monitoring the position and features of contralateral body parts needed to adjust posture or guide motor behavior (Chang et al., 2011). The specific pairwise comparisons revealed distinct differences in fractional amplitude of low-frequency fluctuations (fALFF) among the groups. High-level endurance athletes exhibited significantly higher fALFF in the right precuneus compared to the control group, and significantly higher fALFF in both the left medial superior frontal gyrus and right precuneus compared to ordinary-level endurance athletes. Ordinary-level endurance athletes, in turn, had significantly higher fALFF in the right prefrontal cortex and right parahippocampal gyrus compared to the control group. Additionally, ordinary-level endurance athletes showed significantly higher fALFF in the posterior part of the left cingulate gyrus than high-level endurance athletes, while the control group demonstrated significantly higher fALFF in the left medial frontal lobe compared to ordinary-level endurance athletes, and higher fALFF in the bilateral inferior temporal gyri compared to high-level endurance athletes. These differential brain regions closely align with the default mode network, which includes areas such as the precuneus, posterior cingulate gyrus, medial prefrontal cortex, and inferior parietal lobule (Raichle et al., 2001). The precuneus and medial prefrontal cortex are highly involved in self-related episodic memory (Dorfel et al., 2009). In addition, studies have demonstrated that exercise interventions can enhance the functional connectivity within the DMN in elderly individuals (Wang et al., 2013). The results of this study further corroborated the differences in the default mode networks between endurance athletes of varying levels and the non-athlete control group. Previous studies have found positive structural plasticity changes (gray matter density) in the posterior parietal cortex in basketball players (Tan et al., 2017) and gymnasts (Huang et al., 2015). This study is the first to find positive functional plasticity characteristics in the posterior parietal cortex in endurance athletes. The angular gyrus is part of the inferior parietal lobule and is also involved in the composition of the default network (Raichle et al., 2001). In addition, the inferior parietal lobule is responsible for integrating information from the body and processing external information about objects (Naito and Ehrsson, 2006). The parietal cortex is closely related to spatial awareness (Behrmann et al., 2004). The superior parietal lobule is involved in motion perception (Vaina et al., 2001) and bimanual movements (Wenderoth et al., 2004). The left parietal lobe plays a crucial role in various cognitive and motor functions, including visuospatial attention, memory, mathematical cognition (Uddin et al., 2010), intention to perform specific motor actions (Colby and Goldberg, 1999), and movement planning (Andersen et al., 1997). The left inferior parietal lobule is particularly involved in mental arithmetic, which requires memory and attentional resources, as well as in integrating time and space for collision judgments (Assmus et al., 2003). It also integrates internal bodily information and processes external information about objects (Naito and Ehrsson, 2006). Notably, high-level golfers exhibit increased gray matter volume in the posterior parietal cortex (Jäncke et al., 2009), and gray matter density in the medial parietal lobe has been shown to increase following a brief period of juggling training (Scholz et al., 2009).

In conclusion, functional reorganization of brain regions associated with the default network was observed in average-level endurance runners, potentially linked to their extended training. From a practical perspective on optimizing brain function through exercise, engaging in 1 to 4 years of endurance training appears to be an effective and feasible method for enhancing default network functionality. This relatively brief training period can facilitate positive reorganization of brain function and lead to significant improvements. Ongoing endurance training not only supports the optimization of the default network but also contributes to notable gains in cognitive and emotional domains. Thus, moderate endurance exercise can improve default network function in a relatively short duration and offers practical benefits for overall brain function enhancement.

### 4.3 Limitations

While the expert-novice paradigm provides a practical alternative to the resource-intensive demands of longitudinal studies, it is not exempt from the inherent limitations of cross-sectional research, notably the inability to establish definitive causal relationships. The observed differences in brain structure and functional activity between groups may not be solely attributable to varying levels of endurance training; alternative explanations, such as genetic predispositions, could also account for the superior brain plasticity observed in elite endurance athletes. As such, the findings of this study should be considered preliminary. Future research should incorporate longitudinal designs to track changes in brain structure and function over time in relation to endurance training. Such an approach would enhance our understanding of the causal relationship between long-term endurance training and brain plasticity.

## 5 Conclusion

Adaptive benefits exhibit different characteristics across different endurance levels. High-level endurance athletes exhibit pronounced enhancements in gray matter volume and functional activity in regions associated with memory, motor control, and sensory processing. While moderate-level athletes demonstrate distinct functional reorganization in the default mode network and cerebellum.

## Data Availability

The raw data supporting the conclusions of this article will be made available by the authors, without undue reservation.

## Footnotes

^1^ http://dbm.neuro.uni-jena.de/vbm

## References

[ref1] Andersen R. A. Snyder L. H. Bradley D. C. Xing J. (1997). Multimodal representation of space in the posterior parietal cortex and its use in planning movements. Annu. Rev. Neurosci. 20, 303–330. doi: 10.1146/annurev.neuro.20.1.303 9056716

[ref2] Ashburner J. Friston K. J. (2000). Voxel-based morphometry—the methods. NeuroImage 11, 805–821. doi: 10.1006/nimg.2000.0582, PMID: 10860804

[ref3] Assmus A. Marshall J. C. Ritzl A. Noth J. Zilles K. Fink G. R. (2003). Left inferior parietal cortex integrates time and space during collision judgments. NeuroImage 20, S82–S88. doi: 10.1016/j.neuroimage.2003.09.025, PMID: 14597300

[ref4] Behrmann M. Geng J. J. Shomstein S. (2004). Parietal cortex and attention. Curr. Opin. Neurobiol. 14, 212–217. doi: 10.1016/j.conb.2004.03.012 15082327

[ref5] Bullmore E. Sporns O. (2009). Complex brain networks: graph theoretical analysis of structural and functional systems. Nat. Rev. Neurosci. 10, 186–198. doi: 10.1038/nrn2575 19190637

[ref6] Burdette J. H. Laurienti P. J. Espeland M. A. Morgan A. Telesford Q. Vechlekar C. D.. (2010). Using network science to evaluate exercise-associated brain changes in older adults. Front. Aging Neurosci. 2:23. doi: 10.3389/fnagi.2010.00023, PMID: 20589103 PMC2893375

[ref7] Chaddock L. Erickson K. I. Prakash R. S. Kim J. S. Voss M. W. Vanpatter M.. (2010). A neuroimaging investigation of the association between aerobic fitness, hippocampal volume, and memory performance in preadolescent children. Brain Res. 1358, 172–183. doi: 10.1016/j.brainres.2010.08.049, PMID: 20735996 PMC3953557

[ref8] Chang Y. Lee J.-J. Seo J.-H. Song H.-J. Kim Y.-T. Lee H. J.. (2011). Neural correlates of motor imagery for elite archers. NMR Biomed. 24, 366–372. doi: 10.1002/nbm.1600, PMID: 22945291

[ref9] Colby C. L. Goldberg M. E. (1999). Space and attention in parietal cortex. Annu. Rev. Neurosci. 22, 319–349. doi: 10.1146/annurev.neuro.22.1.319 10202542

[ref10] Di Paola M. Caltagirone C. Petrosini L. (2013). Prolonged rock climbing activity induces structural changes in cerebellum and parietal lobe. Hum. Brain Mapp. 34, 2707–2714. doi: 10.1002/hbm.22095, PMID: 22522914 PMC6870153

[ref11] Di X. Zhu S. Jin H. Wang P. Ye Z. Zhou K.. (2012). Altered resting brain function and structure in professional badminton players. Brain Connect. 2, 225–233. doi: 10.1089/brain.2011.0050, PMID: 22840241 PMC3621728

[ref12] Dorfel D. Werner A. Schaefer M. von Kummer R. Karl A. (2009). Distinct brain networks in recognition memory share a defined region in the precuneus. Eur. J. Neurosci. 30, 1947–1959. doi: 10.1111/j.1460-9568.2009.06973.x, PMID: 19895564

[ref13] Erickson K. I. Prakash R. S. Voss M. W. Chaddock L. Hu L. Morris K. S.. (2009). Aerobic fitness is associated with hippocampal volume in elderly humans. Hippocampus 19, 1030–1039. doi: 10.1002/hipo.20547, PMID: 19123237 PMC3072565

[ref14] Fine E. J. Ionita C. C. Lohr L. (2002). The history of the development of the cerebellar examination. Neurology 22, 375–384. doi: 10.1055/s-2002-36759 12539058

[ref15] Gorham L. S. Jernigan T. Hudziak J. Barch D. M. (2019). Involvement in sports, hippocampal volume, and depressive symptoms in children. Biol. Psychiatry Cogn. Neurosci. Neuroimaging 4, 484–492. doi: 10.1016/j.bpsc.2019.01.011 30905689 PMC6500760

[ref16] Greicius M. D. Supekar K. Menon V. Dougherty R. F. (2009). Resting-state functional connectivity reflects structural connectivity in the default mode network. Cereb. Cortex 19, 72–78. doi: 10.1093/cercor/bhn059, PMID: 18403396 PMC2605172

[ref17] Hardwick R. M. Rottschy C. Miall R. C. Eickhoff S. B. (2013). A quantitative meta-analysis and review of motor learning in the human brain. NeuroImage 67, 283–297. doi: 10.1016/j.neuroimage.2012.11.020, PMID: 23194819 PMC3555187

[ref18] Huang R. Lu M. Song Z. Wang J. (2015). Long-term intensive training induced brain structural changes in world class gymnasts. Brain Struct. Funct. 220, 625–644. doi: 10.1007/s00429-013-0677-5, PMID: 24297657

[ref19] Huang H. Wang J. Seger C. Lu M. Deng F. Wu X.. (2018). Long-term intensive gymnastic training induced changes in intra- and inter-network functional connectivity: an independent component analysis. Brain Struct. Funct. 223, 131–144. doi: 10.1007/s00429-017-1479-y, PMID: 28733834

[ref20] Jäncke L. Koeneke S. Hoppe A. Rominger C. Hänggi J. (2009). The architecture of the golfer's brain. PLoS One 4:e4785. doi: 10.1371/journal.pone.0004785, PMID: 19277116 PMC2650782

[ref21] Jia X.-Z. Wang J. Sun H.-Y. Zhang H. Liao W. Wang Z.. (2019). RESTplus: an improved toolkit for resting-state functional magnetic resonance imaging data processing. Sci. Bull. 64, 953–954. doi: 10.1016/j.scib.2019.05.008, PMID: 36659803

[ref22] Jones D. T. Machulda M. M. Vemuri P. McDade E. M. Zeng G. Senjem M. L.. (2011). Age-related changes in the default mode network are more advanced in Alzheimer disease. Neurology 77, 1524–1531. doi: 10.1212/WNL.0b013e318233b33d, PMID: 21975202 PMC3198977

[ref23] Koch W. Teipel S. Mueller S. Benninghoff J. Wagner M. Bokde A. L.. (2012). Diagnostic power of default mode network resting state fMRI in the detection of Alzheimer’s disease. Neurobiol. Aging 33, 466–478. doi: 10.1016/j.neurobiolaging.2010.04.013, PMID: 20541837

[ref24] Maguire E. A. Gadian D. G. Johnsrude I. S. Good C. D. Ashburner J. Frackowiak R. S.. (2000). Navigation-related structural change in the hippocampi of taxi drivers. Proc. Natl. Acad. Sci. U.S.A. 97, 4398–4403. doi: 10.1073/pnas.070039597, PMID: 10716738 PMC18253

[ref25] Manto M. Bower J. M. Conforto A. B. Delgado-Garcia J. M. da Guarda S. N. Gerwig M.. (2012). Consensus paper: roles of the cerebellum in motor control--the diversity of ideas on cerebellar involvement in movement. Cerebellum 11, 457–487. doi: 10.1007/s12311-011-0331-9, PMID: 22161499 PMC4347949

[ref26] Marchetti I. Koster E. H. Sonuga-Barke E. J. De Raedt R. (2012). The default mode network and recurrent depression: a neurobiological model of cognitive risk factors. Neuropsychol. Rev. 22, 229–251. doi: 10.1007/s11065-012-9199-9, PMID: 22569771

[ref27] Martinelli P. Sperduti M. Piolino P. (2013). Neural substrates of the self-memory system: new insights from a meta-analysis. Hum. Brain Mapp. 34, 1515–1529. doi: 10.1002/hbm.22008, PMID: 22359397 PMC6870171

[ref28] Mauk M. D. Medina J. F. Nores W. L. Ohyama T. (2000). Cerebellar function: coordination, learning or timing? Curr. Biol. 10, R522–R525. doi: 10.1016/s0960-9822(00)00584-4 10898992

[ref29] Mechelli A. Price C. J. Friston K. J. Ashburner J. (2005). Voxel-based morphometry of the human brain: methods and applications. Curr. Med. Imaging 1, 105–113. doi: 10.2174/1573405054038726

[ref30] Mevel K. Chetelat G. Eustache F. Desgranges B. (2011). The default mode network in healthy aging and Alzheimer’s disease. Int. J. Alzheimers Dis. 2011:535816. doi: 10.4061/2011/535816, PMID: 21760988 PMC3132539

[ref31] Miller B. R. Hen R. (2015). The current state of the neurogenic theory of depression and anxiety. Curr. Opin. Neurobiol. 30, 51–58. doi: 10.1016/j.conb.2014.08.012 25240202 PMC4293252

[ref32] Naito E. Ehrsson H. H. (2006). Somatic sensation of hand-object interactive movement is associated with activity in the left inferior parietal cortex. J. Neurosci. 26, 3783–3790. doi: 10.1523/JNEUROSCI.4835-05.2006, PMID: 16597731 PMC6674143

[ref33] Park I. S. Lee K. J. Han J. W. Lee N. J. Lee W. T. Park K. A.. (2009). Experience-dependent plasticity of cerebellar vermis in basketball players. Cerebellum 8, 334–339. doi: 10.1007/s12311-009-0100-1, PMID: 19259755

[ref34] Penhune V. B. Steele C. J. (2012). Parallel contributions of cerebellar, striatal and M1 mechanisms to motor sequence learning. Behav. Brain Res. 226, 579–591. doi: 10.1016/j.bbr.2011.09.044 22004979

[ref35] Raichle M. E. MacLeod A. M. Snyder A. Z. Powers W. J. Gusnard D. A. Shulman G. L. (2001). A default mode of brain function. Proc. Natl. Acad. Sci. U.S.A. 98, 676–682. doi: 10.1073/pnas.98.2.676, PMID: 11209064 PMC14647

[ref36] Raichlen D. A. Bharadwaj P. K. Fitzhugh M. C. Haws K. A. Torre G.-A. Trouard T. P.. (2016). Differences in resting state functional connectivity between young adult endurance athletes and healthy controls. Front. Hum. Neurosci. 10:610. doi: 10.3389/fnhum.2016.00610, PMID: 28018192 PMC5147411

[ref37] Schlaffke L. Lissek S. Lenz M. Brüne M. Juckel G. Hinrichs T.. (2014). Sports and brain morphology—a voxel-based morphometry study with endurance athletes and martial artists. Neuroscience 259, 35–42. doi: 10.1016/j.neuroscience.2013.11.046, PMID: 24291669

[ref38] Scholz J. Klein M. C. Behrens T. E. J. Johansen-Berg H. (2009). Training induces changes in white-matter architecture. Nat. Neurosci. 12, 1370–1371. doi: 10.1038/nn.2412, PMID: 19820707 PMC2770457

[ref39] Sheline Y. I. Barch D. M. Price J. L. Rundle M. M. Vaishnavi S. N. Snyder A. Z.. (2009). The default mode network and self-referential processes in depression. Proc. Natl. Acad. Sci. U.S.A. 106, 1942–1947. doi: 10.1073/pnas.0812686106, PMID: 19171889 PMC2631078

[ref40] Shulman G. L. Corbetta M. Buckner R. L. Fiez J. A. Miezin F. M. Raichle M. E.. (1997). Common blood flow changes across visual tasks: I. Increases in subcortical structures and cerebellum but not in nonvisual cortex. J. Cogn. Neurosci. 9, 624–647. doi: 10.1162/jocn.1997.9.5.624, PMID: 23965121

[ref41] Tan X.-Y. Pi Y.-L. Wang J. Li X.-P. Zhang L.-L. Dai W.. (2017). Morphological and functional differences between athletes and novices in cortical neuronal networks. Front. Hum. Neurosci. 10:660. doi: 10.3389/fnhum.2016.00660, PMID: 28101012 PMC5209359

[ref42] Uddin L. Q. Supekar K. Amin H. Rykhlevskaia E. Nguyen D. A. Greicius M. D.. (2010). Dissociable connectivity within human angular gyrus and intraparietal sulcus: evidence from functional and structural connectivity. Cereb. Cortex 20, 2636–2646. doi: 10.1093/cercor/bhq011, PMID: 20154013 PMC2951845

[ref43] Vaina L. M. Solomon J. Chowdhury S. Sinha P. Belliveau J. W. (2001). Functional neuroanatomy of biological motion perception in humans. Proc. Natl. Acad. Sci. U.S.A. 98, 11656–11661. doi: 10.1073/pnas.191374198, PMID: 11553776 PMC58785

[ref44] Wang B. Fan Y. Lu M. Li S. Song Z. Peng X.. (2013). Brain anatomical networks in world class gymnasts: a DTI tractography study. NeuroImage 65, 476–487. doi: 10.1016/j.neuroimage.2012.10.007, PMID: 23073234

[ref45] Wenderoth N. Debaere F. Sunaert S. van Hecke P. Swinnen S. P. (2004). Parieto-premotor areas mediate directional interference during bimanual movements. Cereb. Cortex 14, 1153–1163. doi: 10.1093/cercor/bhh075, PMID: 15142955

[ref46] Wenzel U. Taubert M. Ragert P. Krug J. Villringer A. (2014). Functional and structural correlates of motor speed in the cerebellar anterior lobe. PLoS One 9:e96871. doi: 10.1371/journal.pone.0096871, PMID: 24800742 PMC4011948

[ref001] Yang F. Jia W. Kukun H. Ding S. Zhang H. Wang Y. (2022). A study of spontaneous brain activity on resting-state functional magnetic resonance imaging in adults with MRI-negative temporal lobe epilepsy. Neuropsychiatr. Dis. Treat. 18:1107. doi: 10.2147/NDT.S366189, PMID: 35677937 PMC9170234

[ref47] Zhang K. Jan Y.-K. Liu Y. Zhao T. Zhang L. Liu R.. (2022). Exercise intensity and brain plasticity: what’s the difference of brain structural and functional plasticity characteristics between elite aerobic and anaerobic athletes? Front. Hum. Neurosci. 16:757522. doi: 10.3389/fnhum.2022.757522, PMID: 35273485 PMC8901604

[ref002] Zhao S. Du Y. Zhang Y. Wang X. Xia Y. Sun H.. (2023). Gray matter reduction is associated with cognitive dysfunction in depressed patients comorbid with subclinical hypothyroidism. Front. Aging Neurosci. 15:1106792. doi: 10.3389/fnagi.2023.1106792, PMID: 36845662 PMC9945283

[ref48] Zou Q.-H. Zhu C.-Z. Yang Y. Zuo X.-N. Long X.-Y. Cao Q.-J.. (2008). An improved approach to detection of amplitude of low-frequency fluctuation (ALFF) for resting-state fMRI: fractional ALFF. J. Neurosci. Methods 172, 137–141. doi: 10.1016/j.jneumeth.2008.04.012, PMID: 18501969 PMC3902859

